# Hospital and Institutional News

**Published:** 1916-08-26

**Authors:** 


### HOSPITAL AND INSTITUTIONAL NEWS.

### HOSPITALS AND THE NATIONAL RELIEF FUND.

Exceptional pressure renders it impossible for us to publish this week a further instalment of the views of provincial and other hospital authorities on this subject. Some hospital committees, like that of the Pendlebury Children's Hospital, have considered the proposal of a grant from the National Relief Fund, and have come to the conclusion that they have no grounds for making any claim on this Fund. The majority of hospitals appear to take an entirely opposite view, which is that of Mr. Danvers Power in his letter to the *Times*. It will be a useful service to the hospitals and the National Relief Fund to have this matter threshed out and determined one way or the other. We shall therefore revert to the subject and deal with it with this end in view. Meanwhile, unless he is shooting grouse or otherwise enjoying himself, we should welcome the elaboration by Mr. Danvers Power of his ideas.

### A WONDERFUL WEEK FOR HOSPITAL LEGACIES.

During the past week particulars are announced of two wills, in each of which the bequests to hospitals run into a total of six figures. One of these wills bestows its benefits chiefly upon institutions in London and Surrey, the other mainly upon Glasgow and the West of Scotland. Thus by the will of Mr. Leopold Salomons, of Norbury Park, Dorking (who some time ago bought Box Hill and presented it to the nation), three institutions receive £1,000 each; these are the Surrey Convalescent Home at Seaford, the Seaside Convalescent Home, Seaford, and the Leatherhead Cottage Hospital. Besides these specific legacies, the residue of the estate, which will apparently amount eventually to about £100,000, is left as to one-fourth to such blind asylums and ophthalmic hospitals in the City of London and the counties of London and Surrey as shall be agreed upon by Mrs. Salomons and three trustees; and the remaining three-fourths is to be similarly distributed among charitable institutions in the same territorial area, not being blind asylums or ophthalmic hospitals. Presumably a large part, if not the whole, of this munificent bequest will reach the voluntary hospitals, or some of them.

### £165,000 FOR SCOTTISH INSTITUTIONS.

Scotland comes off even better than England, for under the will of Miss Mary Hamilton, of Kelvinside, Glasgow, and Ascog, Bute, a total of £165,000 is distributed. The charities which profit most by Miss Hamilton's bequest are the Merchants' House, of Glasgow, which receives £40,000 to endow Hamilton's Mortification Pensions and Precepts, and the Western Infirmary, Glasgow, to which are allotted £30,000 for a "Hamilton Ward," the sum of £7,500 for ordinary purposes, and a further £1,000 for the Samaritan Society. The Glasgow Royal Infirmary gets £10,000 for ordinary purposes, £1,000 for the Dorcas Society, and £1,000 for the Glasgow Ophthalmic Institution (which is the ophthalmic department of the Royal Infirmary). The Victoria Infirmary, Glasgow, receives £7,000; the Glasgow Hospital for Sick Children, £7,500; the Association for the Relief of Incurables, £7,500; the Colquhoun Trust for Incurables, £2,500; the Glasgow Hospital for Diseases of the Ear, Nose, and Throat, £1,000; the Glasgow Eye Infirmary, £1,000; the Glasgow Blind Asylum, £1,000; the Deaf and Dumb Institution, £2,000; the Glasgow and West of Scotland Convalescent Seaside Home, Dunoon, £2,500; the Glasgow Convalescent Homes, Lenzie, £2,000; the Glasgow Institution for Orphan and Destitute Girls, £2,000; three charities for orphans and for the sick poor, £1,000 each; the Royal Infirmary, Edinburgh, £7,500; the Royal Edinburgh Hospital for Incurables, £5,000; the Royal Society for Home Relief to Incurables, Edinburgh, £1,000; the Sibbald Pensions for Relief of Incurables, £1,000, and various other charities, the balance of £21,000. Truly a munificent list of benefactions.

### A PICTURE GALLERY IN A HOSPITAL.

By the will of Mrs. Jane Emma Praed, of Patcham, Sussex, who died on June 18 last, leaving estate of the gross value of £7,896, of which £4,879 is net personalty, the sum of £300 is bequeathed to the Brompton Hospital for Consumption and Diseases of the Chest. Further, she also left to that hospital a collection of pictures, directing that they shall be hung in the hospital and not sold; and suggesting that the most valuable of them shall be placed in a room, for admission to which, though free to patients, a small fee shall be charged to the public for the benefit of the hospital. Without knowing the details about these pictures, to which the testatrix evidently assigned great interest and value, it is difficult to judge whether the Brompton Hospital is likely to derive much profit from their exhibition; unless there are pictures of outstanding merit and importance among them it is possible that they may become somewhat of a white elephant. The exhibition of pictures for profit means devoting a room to the purpose, and also absorbs the energies of attendants, turnstile-keepers, and so forth. The total value which is set upon the estate would appear to preclude the possibility of these pictures being of first-rate artistic importance. The hospital authorities have not yet come to a decision as to the disposal of these pictures.

### RELATIONS AND THE WHEREABOUTS OF WOUNDED SOLDIERS.

A correspondent, writing in the columns of the *Birmingham Post*, complains that the returns and reports from the military hospitals are "irremediably in arrear," with the result that "numbers of people all over the country are vainly seeking information." Certainly, if the recent pressure on the hospitals is to be maintained, the question of increased staffs will have to be faced. The remedy suggested for the delays complained of is that in each city a list of all the casualties in the local hospitals should be placed and made available for inspection. It is further suggested that municipalities or military medical authorities should mutually exchange lists of patients according to their places of residence. Relatives of wounded men are clearly entitled to prompt information, but the returns might be expedited without the general system of intelligence here proposed, for people would always prefer direct information to making visits to a local centre on the chance of seeing the name in which they were interested entered in the local exchange list. Moreover, the British Red Cross Society has an efficient department undertaking the exact duty suggested, to which application can be made by friends of wounded soldiers of whom trace has been lost.

### A STARTLING SUGGESTION.

A curious and somewhat cryptic statement was made before a recent meeting of the Public Health Committee at Bristol by Dr. Davies, the medical officer of health. The committee was considering three recent cases of illness at Bristol which have been proved to be mild cases of bubonic plague. Infected rats have been discovered, and Dr. Davies was asked whether he was propounding the theory that a certain rat which has been caught had been deliberately infected, to which he replied: "I should not be surprised. It is quite possible. I may have a further statement to make to the committee upon that point when I have made further investigations." The obvious suggestion of these startling sentences is that German agents have been trying to start an epidemic of plague in this country by introducing infected rats into one of our seaports. Such a proceeding is, of course, quite contrary to International Law; but then so is the poisoning of wells and many other foul devices of which the enemy have been proved guilty during this war. If this is indeed what has happened, medical officers of health, especially at all seaports, will have to be doubly vigilant in tracing and limiting all suspicious epidemics.

### THE IMPORTATION OF INFANTILE PARALYSIS.

In this connection it is also important that all possible precautions shall be taken against the spread of the epidemic of poliomyelitis (infantile paralysis), which is at present raging in New York. The difficulty is that the presumed micro-organism of this disease has not yet been discovered, and the method by which an epidemic spreads is quite unknown. The hypothesis that flies, particularly stable flies, act as disseminators of infection has been supported by some authorities, and there are facts which are in its favour; but nothing approaching proof of this view has been secured, and just now there is a tendency to look elsewhere for the infecting agency. At all events, the New York epidemic is a very serious one: the death-rate has reached the unusual height of 25 per cent. The disease, though it principally affects children under five, is not confined to them, and may attack adults. In view of the congregation of large bodies of soldiers under training it is vital that the infection shall not reach this country if it can be prevented from so doing. In America strict quarantine regulations have been adopted for travellers leaving infected areas; and it is being suggested that similar regulations—including ten days' isolation—shall be enforced on passengers coming from New York and other infected areas, and that thorough disinfection shall be practised in all cases in which a shadow of doubt may exist.

### ON TOUR AMONG THE WOUNDED.

Under the auspices of the Birmingham branches of the British Red Cross Society and the Navy League, the Alexandra Musical Society of Birmingham has been paying a visit to North Wales during August. The plan apparently originated at the request of a number of Birmingham men in hospitals and in the training camps of North Wales, and therefore the Alexandra Musical Society of Birmingham, which has done much for the wounded since the outbreak of war, has undertaken quite a tour of entertainment. The series began at the Red Cross Hospital, Rhyl, on August 12, and extended over a week, when convalescent hospitals and training camps at Llandudno, Deganwy, Conway, and Bangor were visited. We understand that entertainments are given voluntarily at war hospitals and training camps upon application to Mr. E. C. Thomas, at the Navy League Offices, Queen's College, Paradise Street, Birmingham. So far more than 150 entertainments have been given, and it seems that the district covered is anywhere within sixty-five miles of Birmingham.

### ECONOMY IN HOSPITAL GARDENS.

The Royal Horticultural Society has prepared a series of pamphlets for the assistance of amateur and cottage gardeners with a view to the encouragement of economy by increasing the home production of vegetables and fruit, and maintaining existing gardens inexpensively during the war. Many of those hospital managers whose institutions possess gardens or grounds that have not been properly developed would do well to study these pamphlets. The one which covers the general principles and contains the most useful collection of practical hints is entitled "Economy in the Garden," price threepence. Its instructions are detailed and practical, and if followed out would increase the yield or save the expense of maintaining the grounds of most institutions which possess them. Copies may be obtained from the Royal Horticultural Society's Offices, Vincent Square, Westminster, together with the society's other pamphlets on special points of practical horticulture.

### A RECORD ADMISSION.

There is nothing worthy of comment in the mid-August issue of the 1st Eastern General Hospital *Gazette* except the statement that a succeeding number will be devoted to a detailed account of the King's recent visit to Cambridge. Prizes have been offered for the best photographs taken on the occasion, and the winning photographs will be published in the next issue. The page of statistics records the admission of 2,434 patients during July, which, it is hardly surprising to learn, eclipses all previous figures. An arts and crafts exhibition has been held, the proceeds of which, amounting to three figures, are to provide winter entertainments; but the only information furnished in this issue is the balance-sheet. In so large an institution a *Gazette* should be more varied, and indeed more ambitious, than that of the 1st Eastern General Hospital appears to be.

### AN UNUSUAL CASE.

There is one curious item in the expenditure accounts of the Royal Cornwall Infirmary, Truro, which deserves mention, as it is very unusual in English hospital accounts. This is a sum of £52 entered as incurred for the maintenance of a leper at Liskeard. It would be interesting to know how the Royal Cornwall Infirmary came to be responsible for such a case, and what arrangements have been adopted for its proper care. Such cases are, fortunately, so rare that Mr. J. C. R. Crewes, the secretary, might make the most of the opportunity afforded by the hospital's readiness to undertake exceptional cases to interest the public still further in hospital activities.

### ENTERTAINING WOUNDED AT ENGLISH PORTS.

A year ago the Port of London established a scheme for giving to the wounded a series of river trips up and down the Thames in their steamer the *Conservator*. Their example has been followed at Liverpool, where a service of Saturday afternoon excursions for men in the local hospitals has been organised with the co-operation of the principal lines. For instance, on August 12 last the White Star Line, following the Cunard, invited 500 wounded men from eleven hospitals to take an afternoon trip on their tender, the *Magnetic*. A military band entertained and a number of ladies waited on the guests, who were looked after in the matter of refreshments by the victualling superintendent of the line. The scheme originated locally with Mr. C. R. Cheshire and Mr. J. B. Burrows, and while the service should continue as long as the warm weather permits, other ports might well follow the example of London and Liverpool.

### HOSPITAL ENTERPRISE IN ABERDEEN.

The organisation on a large scale of fêtes on behalf of hospitals has been more characteristic of London than of the provinces, but a really ambitious gala day which deserves attention has lately been held at Aberdeen. The scheme, which was started a year ago for the benefit of the infirmary, consists not only of the now familiar Badge Day, held in the city, but also of an elaborate fête in Duthie Park, in which the chief attractions were an exhibition of horse-trotting in sulkies, a jumping competition, and a display of driving by first-prize winners. Over £1,000 was raised by the fête, the Badge Day, and a special Sunday concert. All the hospitals in the city, it is understood, will benefit by these undertakings, which form one of the most ambitious series of charity festivities undertaken in the provinces for hospital purposes.

### HOW TO RECOUP AN ANNUAL SUBSCRIPTION.

Canon J. T. Fowler, a St. Thomas's Hospital man, who qualified in 1856, and afterwards became house surgeon at Bradford Infirmary until he left in 1861 with a view to taking holy orders, is reported to have lately told the following tale. Among his recollections of the infirmary at Bradford is one of a constant visitor who used to make his appearance at the hospital every Sunday about the middle of the day. As time went on the visitor used to be accompanied by his wife and daughter; and as they all took their Sunday luncheon at the infirmary it was estimated that the gentleman recouped his annual subscription in this way. This story, which is quoted among other recollections in the *Yorkshire Observer*, leads us to hope that Canon Fowler may be induced to revive all his memories of a house surgeon's life fifty years ago, and thus add to the value of an original career which has led him, like very few others, to exchange medicine for holy orders.

### A WORKING MAN'S CONVALESCENT HOME.

The third of four convalescent homes erected by the Working Men's Club and Institute Union has been opened at Grange-over-Sands, Lancashire, by Mr. C. Duncan, M.P. The aim of the Union is to establish four of these homes in the four corners of England, so that one may be accessible to any of the 520,000 members. The first home was opened at Pegwell Bay in 1894, and altogether a sum of £14,000 has been spent in extending it. The second home was opened at Saltburn in 1909, while the latest, in Lancashire, is also the most ambitious, for its total cost will be some £23,000, and it provides accommodation for sixty-six residents. The fourth home will be built, after the war, at Langland Bay, near Swansea, South Wales. The homes are all on a self-supporting basis, and are managed by the Union for the associated clubs. The homes in design resemble hotels more than ordinary convalescent homes, with separate bedrooms instead of wards, a liberal supply of recreation rooms, and servants rather than medical men or nurses on the staff. As a special type of convalescent home they have their own interest. The architect of the latest building, at Grange-over-Sands, is Mr. William Wadman, of New Cross, S.E.

### THE HOSPITAL SATURDAY FUND IN LEICESTER.

At the half-yearly meeting of the delegates of this society, held at the Royal Infirmary, it was reported that twenty-five firms, employing 1,013 workpeople, had been added to membership since the last meeting. One hundred and ninety-six male members and 176 female members had received convalescent benefit during the half-year, a total of 372 beneficiaries, as compared with 482 in the corresponding period of last year. One hundred and thirty-three military patients had been received in the Desford Convalescent Home of the Fund, which the members placed at the disposal of the military authorities as an auxiliary hospital. The expenditure of the society was £2,600—£1,443 for the Desford Home, £834 for the Swithland Home, and £321 for maintenance in other homes. £2,500 had been invested in 5 per cent. Exchequer Bonds, making £5,000 thus lent to the Government. The adoption of the report was moved by Mr. S. A. Gimson, who presided. Other speakers were Mr. J. Gipson Clarke, Mr. T. Fielding Johnson, junr., the chairman of the board of the infirmary, and Colonel C. J. Bond, vice-chairman, the two latter acknowledging the very real help which the Saturday Fund had been in the provision of a substantial portion of the income of the infirmary. At the close of the meeting the delegates, to the number of 250, were entertained to tea by the kindness of Mr. Gipson Clarke, the vice-president.

### LODGINGS IN THE WORKHOUSE.

The Guardians, and, indeed, Poor-Law buildings and administration, are continually being made responsible for new duties, and therefore it will hardly come as a surprise to see the announcement that, owing to the scarcity of their usual class of inmate, lodgings are being given in workhouses to prosperous citizens. Among these are persons employed in war hospitals, and the Paddington Guardians are reported to have undertaken to lodge forty hospital workers at a charge of 3s. 2d. a head per week, full board being given on Sundays. Munition workers have already been housed by various Boards of Guardians, and now the Local Government Board is understood to be arranging with local workhouses for the accommodation of those conscientious objectors who are to be employed on road-making. We are inclined to think that this method of using the workhouse is to be welcomed as one more step in breaking down the barrier which cuts off the life of the "pauper" from his fellows, and therefore another move in the direction of the humanisation of the Poor-Law.

### AN INNOVATION IN SCOTLAND.

The Royal Maternity Hospital, Glasgow, has decided to open a lady almoner's department, and it is stated that this will be the first Scottish hospital to employ a trained lady almoner. The governors have decided to run the department on similar lines to those existing in the lady almoner's department at St. Thomas's Hospital, whose maternity work was especially commended in a recent report of the Local Government Board. Miss P. M. Lyall, late assistant almoner at St. Thomas's Hospital, has been appointed to the post, and will start work in Glasgow at the beginning of September. If our information that this lady will be the first pioneer of almoner's work in Scotland is correct, it would be of interest to know why Scottish institutions hitherto have not found it advisable to adopt the English practice; and also the reasons that have induced this Glasgow institution at length to do so.

### A SUCCESSFUL APPEAL.

The British Home and Hospital for Incurables, Streatham, have been successful in an appeal for exemption from military service for their head-attendant. The secretary explained that the hospital was used as an auxiliary medical establishment by the War Office, but refused any help in the way of male or female nurses. This man, therefore, was indispensable, and he was successful in getting the Tribunal at Lambeth to agree with him, for they granted conditional exemption so long as the man was in his present occupation. As a general rule, the Tribunals have dealt very fairly with the applications for exemption for members of the general staffs of the hospitals and infirmaries of London, though occasionally they have had to be strict in cases where there has been an attempt to obtain exemption for men whose places could be easily filled by either women, men over military age, or soldiers discharged from the Forces.

### THE MUTABILITY OF THE HOSPITAL STAFF.

The appointment of a new secretary to the City of London Lying-in Hospital, the titular secretary, Mr. Spencer Johnson, having no doubt come to the conclusion that at times the sword is mightier than the pen, brings home to us the thought that in institutional life the war will effect many changes. Of those officers who set forth in the early stages—doctors, matrons, secretaries, sisters, and others—unhappily we know a number can never resume their civil duties. Others who looked forward to dropping back into the same groove at the end of a few months have been borne by the flux of events into entirely different channels. Even in ordinary times the mutability of the *personnel* of the hospital staff is a commonplace, best realised by looking at a comparatively recent photographic group which reveals how brief, except in respect of certain offices, is the duration of most hospital appointments; but in these times this condition is much more apparent, and a few months may change the personality of the whole staff from the stores-boy to the matron. The gleaners are still busy, and in a few weeks or months many more familiar figures will be absent and their work must be provided for out of the wonderful store of reserve activity already so steadily drawn upon. An idea is being discussed in hospital circles, in reference to the calling up of the younger secretaries, which has many useful points. It is that a remaining secretary shall, in addition to his own work, serve a secretaryless hospital in a consultative capacity, attending committee meetings, occasionally visiting his adopted hospital, and holding himself available to advise on points of difficulty arising on the administrative side.

### THE WINCHESTER COUNCIL AND THE HAMPSHIRE COUNTY HOSPITAL.

After a good deal of discussion, the Winchester Town Council adopted the recommendation of the General Purposes Committee that twenty guineas annually should be subscribed to the funds of the Royal Hampshire County Hospital. Objections were raised on the score of the legality of such an expenditure, but the Town Clerk stated that, as an employer of labour, the Corporation can subscribe to a hospital in which its own employees are treated; and, further, that the President of the Local Government Board raised no objection to the proposal. There was some desultory opposition, but the Mayor made a firm speech in support of the resolution, which was carried by fourteen votes to six. The sum voted is not large, but as a recognition for the first time of the benefits which the Corporation employees receive from the County Hospital, it at least establishes a precedent of the right sort in Winchester. In the North of England there is less hesitation in voting public money to hospitals when good cause is shown.

### THE INSURANCE OF HOSPITAL BUILDINGS.

A discussion at a recent meeting of the Cork Joint Hospital Board suggests the existence of an insurance "ring" in Ireland which is distinctly prejudicial to public and institutional interests. The point arose on a proposal that the insurance on the buildings and furniture, for which the Joint Board is responsible, should be increased by £9,900, owing to their enhanced value since the last valuation. Five companies tendered, four of them at five shillings per cent., the other at two shillings and threepence. Although the latter company was stated to be quite stable and reputable, it was decided to divide the insurance among the other four, for the reason that these companies can re-assure, whereas the fifth company cannot do so. In other words, there is a combine amongst the insurance companies to keep the rates up; and any company which tries to cut the rates is penalised by the refusal of the others to accept re-assurance business with it. We do not know whether anything of the same sort exists in England; but we sincerely hope that this ingenious plan for milking the public is not in existence on this side of St. George's Channel.

### TYPHUS FEVER IN GLASGOW.

The brief outbreak of typhus fever which occurred in Glasgow in July has been the subject of a special report to the Public Health Committee of the City Council by the medical officer of health, Dr. A. K. Chalmers. Eight cases in all were identified, all but two of which were connected with one set of tenements; the other two appear to have been contact cases. From the fact that the first four cases to be recognised began almost simultaneously, it has been inferred that there must have been some precedent source of infection; but the most careful search has failed to trace it. The short duration and limited nature of the epidemic reflect credit upon the public health staff of Glasgow; it is satisfactory that this dirt disease was so speedily detected and investigated. The report presented by Dr. Chalmers pointedly remarks that of the four houses from which cases were notified three were verminous. It would be premature as yet to conclude that the outbreak has been wholly quelled, because the source of it has not been traced, and may conceivably be still active; but there seems to be every hope that this reservation may prove to be academic, and that no more cases will occur.

### A NEW HOSPITAL AT THURGARTON.

A country home for children suffering from consumption has been opened at Thurgarton, under the auspices of the Nottingham Sanatoria and Women Workers' Care Committee. The venture—which is the first of its kind in Nottinghamshire—was made possible through the generosity of Mrs. Upton, who gave the house for the home and a good deal of the furniture. At present six girls and two boys are in residence, but the full complement of twelve will soon be forthcoming. The estimated cost of maintenance is 12s. 6d. per child per week, some small share of which it is hoped the parents will be able to pay. A subscription income of £200 a year will also be required.

### LONDON'S SANATORIUM DEFICIENCIES.

If the statements made at the London Insurance Committee are correct, it is high time there was more adequate provision of sanatorium facilities for insured persons in the Metropolis. It appears that London, with its teeming population, has only 270 sanatorium beds at its disposal, and the Commissioners are seeking to set a limit to the right of insured persons to these benefits. The committee has passed a resolution protesting against this action. At the present moment there are 200 insured consumptives on the committee's waiting list.

### THE LIBERTY OF THE TUBERCULOUS SUBJECT.

At a recent meeting of the Council of the Welsh National Memorial Association at Llandrindod Wells the question of the compulsory detention of phthisical patients was brought forward by one of the members. It is strange to note the storm of opposition that invariably arises whenever this matter is broached. In such deep respect is the sacred "liberty of the subject" held by a large number that any attempts to curtail or destroy what is regarded as an inviolable right of every citizen, in health or in sickness, are certain to be met with hostile criticism or even scornful abuse. And yet these same objectors do not raise a finger against the forcible removal of a patient suffering from one or other of the infectious fevers. If consumption of the "open" type be not so actively contagious as scarlet fever or smallpox, the tubercle bacillus is equally capable of disseminating itself in susceptible soil, there to work out its hidden and malevolent designs. One has only to visit the sick-room of a poor consumptive in the advanced stage in some squalid slum, surrounded by all manner of insanitary belongings, to realise the danger that such a case may be to the community at large. It should not be an insuperable difficulty to provide legislation which would give to local authorities power to act in cases where the risks of infection are clear, and following this would come the provision of suitable accommodation for such patients.

### A DEADLOCK IN PEMBROKESHIRE.

The scheme propounded by the Pembrokeshire County Council for the treatment of tuberculous patients is not meeting with a very favourable reception. Pembroke is the only county which refuses to co-operate in the Welsh National Memorial Association, and the position thus created is full of difficulties. The County Insurance Committee have refused to fall in with Pembroke's own scheme, and the Welsh Insurance Commissioners, being of opinion that the entire question of the treatment of tuberculosis will have to be reconsidered when peace is restored, suggests that Pembroke's scheme shall be shelved temporarily and a provisional arrangement come to without prejudice to future action. The County Council, however, contends that this is trying to force them into the Memorial Association, and a deputation has been appointed to confer with the Insurance Commissioners. The Memorial Association can hardly be expected to continue providing treatment for Pembroke patients when the County Council refuses to pay any part of the expense; and if Pembroke does nothing else, it should make arrangements for the treatment of those tuberculous patients who are not insured persons. In 1914 the tuberculosis death-rate for Pembroke was the highest for any county in England and Wales except one.

### INFANT WELFARE IN DENBIGHSHIRE.

The baby-saving campaign is flourishing greatly and gains new adherents every week. The latest recruit is the Denbighshire County Council, which has become alarmed at two disturbing factors present in its area of administration—the lowness of the birth-rate and the highness of the infant death-rate. While little can be done by the authority in regard to the birth question; efforts are to be put forward to save the babies that are born and ensure their after welfare. Maternity and Infant Welfare Centres are to be established in various parts of the county, and health visitors and local committees appointed. The scheme—towards which Mr. and Mrs. Joseph Davies, of Dinas Powis, have given £200—will be directed by a lady medical officer, whose salary has been fixed at £350 a year.

### THE C.E.T.S. AND THE ARMY.

Since the beginning of the war the Church of England Temperance Society has provided upwards of eighty recreation huts, tents, and clubs for the troops in camp in various parts of England. These huts have been a great help to thousands of our soldiers—including the Canadians at Shorncliffe Camp, to mention but one of the centres of the C.E.T.S. recreation activities. Many more could be provided to meet urgent wants if sufficient funds were forthcoming; and the Bishop of Croydon, the chairman of the Society, is appealing to the public for financial help for this purpose. Though much has been done by this Society, the Y.M.C.A., and other organisations, for the comfort of our defenders, much yet remains undone; and we trust the Bishop of Croydon's appeal will not fall upon deaf ears. Contributions may be addressed to 30 Marsham Street, Westminster.

### COCAINE FOR UNREGISTERED DENTISTS.

We are sorry to see that the Home Secretary has yielded to pressure from several private members of the House of Commons, and has extended—or proposes to extend—the temporary permits which he had already granted to unregistered dentists for the purchase of cocaine preparations; though our disappointment with this spineless docility is tempered by his promise to make further inquiries into the subject. If he will direct these further inquiries towards expert sources of information he will learn—as we stated a fortnight ago—that there are local anæsthetics which are far better suited for dental work than cocaine, and do not possess the terrible disadvantages and risks which attach to the use of that alkaloid. He will also learn that some of his critics and questioners in the House are mistaken in their information concerning the harmlessness of cocaine in the hands of unregistered dentists. Exactly how the latter manage to pull so many wires in politics we are at a loss to explain; but the fact that it is done is quite obvious. We should like to know what the medical men in the House of Commons have been about. Surely it was "up to" them to enlighten their colleagues as to the facts about these drugs; not one of them appears to have opened his mouth.

### RACE SUICIDE AT DERBY.

Some rather remarkable circumstances are shown in high relief in the annual report of Dr. E. A. Brindley, the Derby Medical Officer of Health. Not since 1900—at the time of the South African War—was the marriage-rate (18.2) so high. The birth-rate of 21.7, however, is the lowest on record, while the death-rate of 14.6 has not been equalled during the past eleven years. These statistics, as Dr. Brindley points out, cannot be considered very satisfactory. The infantile mortality is happily low (ninety-four per thousand), and it is also a matter for congratulation that the town has now been free from small-pox for the space of eleven years. The medical officer pays a tribute to the admirable efforts put forth by the "lady substitutes" in his department to cope with increased work.

### THIS WEEK'S DRUG MARKET.

Few remarkable price changes have occurred during the week, and business has continued quiet, which is not surprising in view of the fact that the tendency of prices in the case of several of the more important drugs is in a downward direction. There is still no noteworthy change in the price of cocaine, but the general opinion is that the value will tend downwards. Hypophosphites are dearer. Acetanilide is again rather lower in price, and the same may be said of aspirin. Salicylates are unchanged. Barbitone is dearer. There is no further change to report in bromides, and the market is quiet. While makers' prices of bismuth preparations are unchanged the market is not so strong; hitherto holders of stocks have been able to obtain premiums on makers' prices, but they are not now able to do so. Hexamine still tends downwards in price. Phenacetin continues scarce, and prices are well maintained. Quinine is quiet and unchanged. Carbolic acid is obtainable at slightly lower prices.

